# A cluster randomized trial of a comprehensive intervention nesting family and clinic into school centered implementation to reduce myopia and obesity among children and adolescents in Beijing, China: study protocol

**DOI:** 10.1186/s12889-023-16270-x

**Published:** 2023-07-27

**Authors:** Xinxin Wang, Jiajia Dang, Jieyu Liu, Yunfei Liu, Yaqi Wang, Di Shi, Ziyue Chen, Wen Yuan, Shan Cai, Jie Mi, Pei Xiao, Li Li, Yunwei Fan, Aiyu Gao, Haihua Chen, Lili Zhuang, Zhaocang Yu, Jianhui Li, Dongmei Yang, Gang Yang, Lipo Guo, Yuanyuan Li, Jieyun Song, Jing Li, Jun Ma, Yanhui Dong, Yi Song

**Affiliations:** 1 School of Public Health, Key Laboratory of Environmental Factors and Chronic Disease Control, Ningxia Medical University, No.1160, Shengli Street, Yinchuan, 750004 Xingqing District China; 2 School of Public Health, National Health Commission Key Laboratory of Reproductive Health, Institute of Child and Adolescent Health, Peking University, Beijing, 100191 China; 3 Center for Non-Communicable Disease Management, National Center for Children’s Health, Beijing Children’s Hospital, Capital Medical University, Beijing, 100045 China; 4 Department of Ophthalmology, National Center for Children’s Health, Beijing Children’s Hospital, Capital Medical University, Beijing, 100045 China; 5 Dongcheng Primary and Secondary School Health Care Center, Beijing, China; 6 Beijing Tongzhou District Primar Yand Secondary School Health Carelnstitute, Beijing, China; 7 Tongzhou District Center for Disease Control and Prevention, Beijing, China; 8 Health Education Center forPrimary and Secondary Schools Changping Beijing, Beijing, China

**Keywords:** Children, Adolescents, Myopia, Obesity, Custer randomized trial

## Abstract

**Background:**

Myopia and obesity in children and adolescents have become serious public health problems that endanger public health, especially in China. Unhealthy lifestyle behaviors are environmental drivers of both myopia and obesity. This protocol describes a study to evaluate the effectiveness of “22510SS”, that is 2 h of daytime outdoor activities (‘2’); Limit screen time to no more than 2 h per day (‘2’); Consume at least 5 servings of fruits and vegetables daily (‘5’); Attain 1 h of physical activity daily (‘1’); Consume 0 sugar-sweetened beverages (‘0’); Reasonable sleep duration (‘S’); Regular supervision (‘S’). A school-based, multifaceted intervention strategy for myopia and obesity prevention, and to assess and explore the implementation of “22510SS” with regards to acceptability, feasibility, adoption, usage and maintenance.

**Methods and analysis:**

This study aims to develop a comprehensive intervention strategy "22510SS" based on the socio-ecological model, and A two-arm cluster randomized trial with a parallel-group of a 1:1 allocation ratio in 36 primary and secondary schools to test its evidence-based intervention programs on the effects and implementation of myopia and obesity epidemics in children and adolescents in grades 4 and 7. The primary outcomes will include differences in visual acuity, body mass index, outdoor activity indicators, screen time, fruit and vegetable intake, high-quality protein intake, sugar-sweetened beverage intake, sleep duration, and level of monitoring among children and adolescents. Secondary outcomes will assess the acceptability, feasibility, uptake, use, and maintenance of the intervention. Effects on the primary and secondary outcomes will be analyzed using linear and logistic regression analyses, as well as difference-in-difference analysis, taking into account cluster effects and possible confounding factors. Process assessments will also be conducted through quantitative and qualitative analyses, including acceptability, feasibility, gender, adoption, implementation, and sustainability.

**Discussion:**

This study will evaluate the effectiveness of “22510SS” and examine its implementation in the school-based network nesting family and clinic. Following this intervention study, the integrated intervention program focused on myopia and obesity among children and adolescents have great potential to be implemented in China to promote and support healthy lifestyle behavior change and reduce the risk of myopia and obesity in children and adolescents.

**Trial registration:**

NCT05275959. Registered 23 Mach 2022.

**Supplementary Information:**

The online version contains supplementary material available at 10.1186/s12889-023-16270-x.

## Introduction

Globally, the prevalence of myopia and obesity among children and adolescents has become a serious public health problems concern [1, 2]. In China, over the past 30 years, the incidence of myopia and obesity has risen significantly, with an estimated 180 million myopic and 58.92 million overweight or obese children and adolescents by 2030 [3, 4]. Myopia in adolescence is a growing concern, as it increases the risk of various ocular diseases such as cataracts, glaucoma, macular degeneration, and retinal detachment, which can lead to early visual impairment and even vision loss, affecting academic performance and limiting employment options in adulthood [5–7]. Meanwhile, childhood obesity can result in metabolic abnormalities and early target organ damage, persisting into adulthood and affecting cytokine and hormonal status [8]. This significantly increases the risk of morbidity and mortality, including type 2 diabetes, cardiovascular disease, metabolic disease, and various cancers [8–10]. Currently, a high incidence of myopia and obesity complications in children and adolescents has been observed in cross-sectional studies from Ireland [11] and the Netherlands, [12] showing a positive correlation between the incidence of myopia and body mass index (BMI). Although comprehensive interventions for both myopia and obesity are lacking, there is abundant evidence for individual interventions, and many of these actions overlap [1, 13, 14]. For example, obesity prevention guidelines recommend children consume ≥ 5 servings of fruits and vegetables (‘5’), have ≤ 2 h screen time (‘2’), attain 1 h of physical activity (‘1’), and drink 0 sugar-sweetened beverages (‘0’), commonly known as ‘5–2-1–0’ recommendations [15, 16]. Actually, at the individual level, outdoor activities, [1, 17–22] while a sedentary lifestyle [19, 20], excessive screen time [23, 24], and high-fat diet [25–30] might also contribute to the occurrence of myopia. Therefore, changing 1 h of physical activity as 2 h outdoor time (‘2’), adding 1 serving of high-quality protein daily (‘1’), reasonable sleep duration (‘S’), and regular supervision (‘S’) as intervention package of “22510SS” might work on both myopia and obesity prevention [31].

Interventions targeted towards families have shown some effectiveness in reducing myopia and obesity among children, as parental attitudes and behaviors can have a significant impact [32–35]. At the interpersonal and organizational level, school health education is a crucial factor that can influence myopia and obesity rates [36, 37]. Studies conducted in countries such as Estonia and Russia have found that school health education can effectively lower the prevalence of myopia and obesity by promoting outdoor activities, reducing sedentary behavior, and improving dietary habits [38–40]. At the community and policy level, clinics working together with families and schools play a vital role in mitigating the risks associated with myopia and obesity. Community-based interventions, early detection, and referral to specialist care for further management are essential in identifying children at risk [41–43]. Although the previous researches demonstrate some evidence for the effectiveness of specific myopia or obesity interventions, almost no single intervention will be effective alone, thus, to address the issue comprehensively, it is necessary to integrate existing intervention techniques and summarize “22510SS” by using a social-ecological model (SEM) [44, 45], which might help embed family and clinic into a school centered intervention to reduce myopia and obesity among children and adolescents.

Considering that emerging evidence of the potential impact of a multifaceted approach on myopia or obesity exists, and consolidated intervention is urgently needed to urge myopia and obesity epidemic looming, we will use the RE-AIM framework, which was originally conceived in 1999 to assess and improve the external effectiveness and sustainability of public health interventions from five dimensions of reach, effectiveness, adoption, implementation, and maintenance, [46–48] to assess “22510SS” intervention strategy at the same time to accelerate the implementation and application, and the potential for wider adoption of “22510SS” in other regions of China [49]. The RE-AIM framework ensures regular collection of execution results during the execution process, adjusts the assistance of the assistant team promptly, minimizes the workload, and improves the work efficiency of the organization. After the evaluation completing, an implementation strategy for the evaluation phase will be formulated to prepare for long-term implementation.

## Aims

The purpose of this protocol was to develop a comprehensive intervention strategy, “22510SS” to address the high increase of myopia and obesity in children and adolescents, guided by SEM. The effectiveness and implementation of this intervention were tested using the RE-AIM framework. The main objectives were: (i) to assess the differences in the intervention’s impact on children and adolescents’ visual acuity, body mass index, and behavarial indicators including outdoor activities, screen time, fruits and vegetables, high-quality protein and sugar beverage intake, sleep duration, and supervision. (ii) to evaluate the Reach, Effectiveness, Adoption, Implementation, and Maintenance of the “22510SS” intervention strategy using the RE-AIM framework as a guide.

## Design and methods

**Fig. 1:**
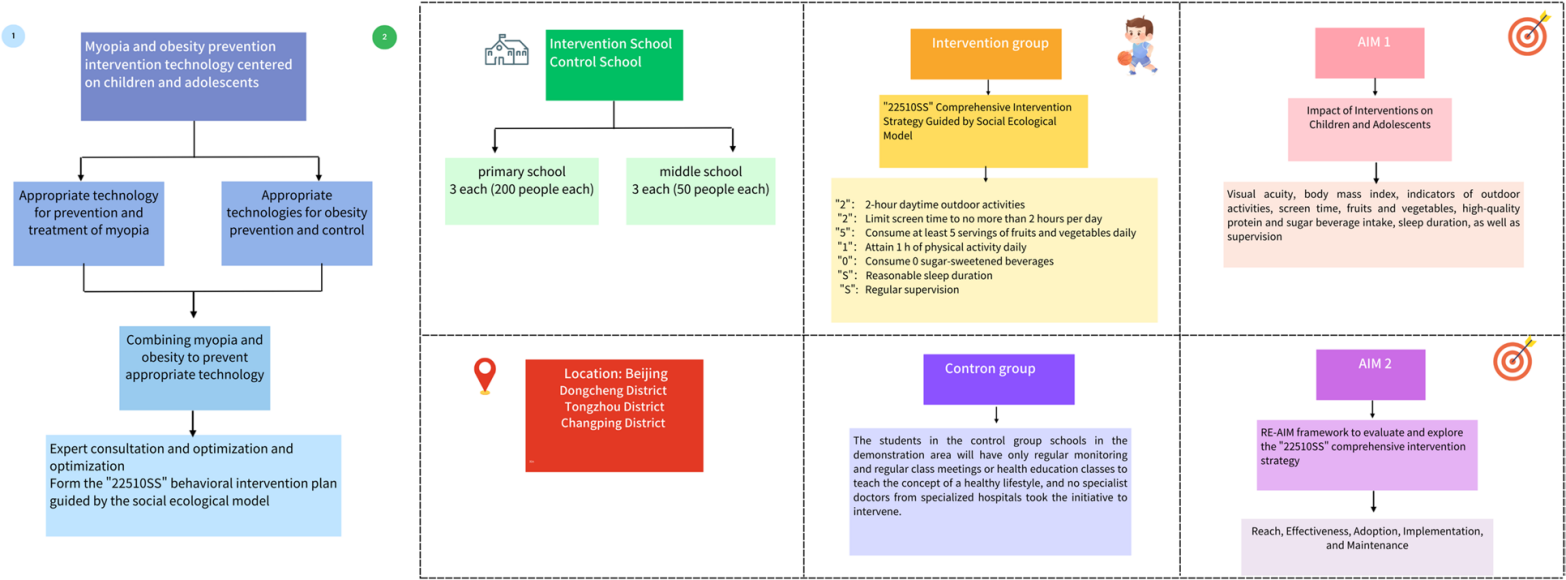
Trial implementation process

**Fig. 2:**
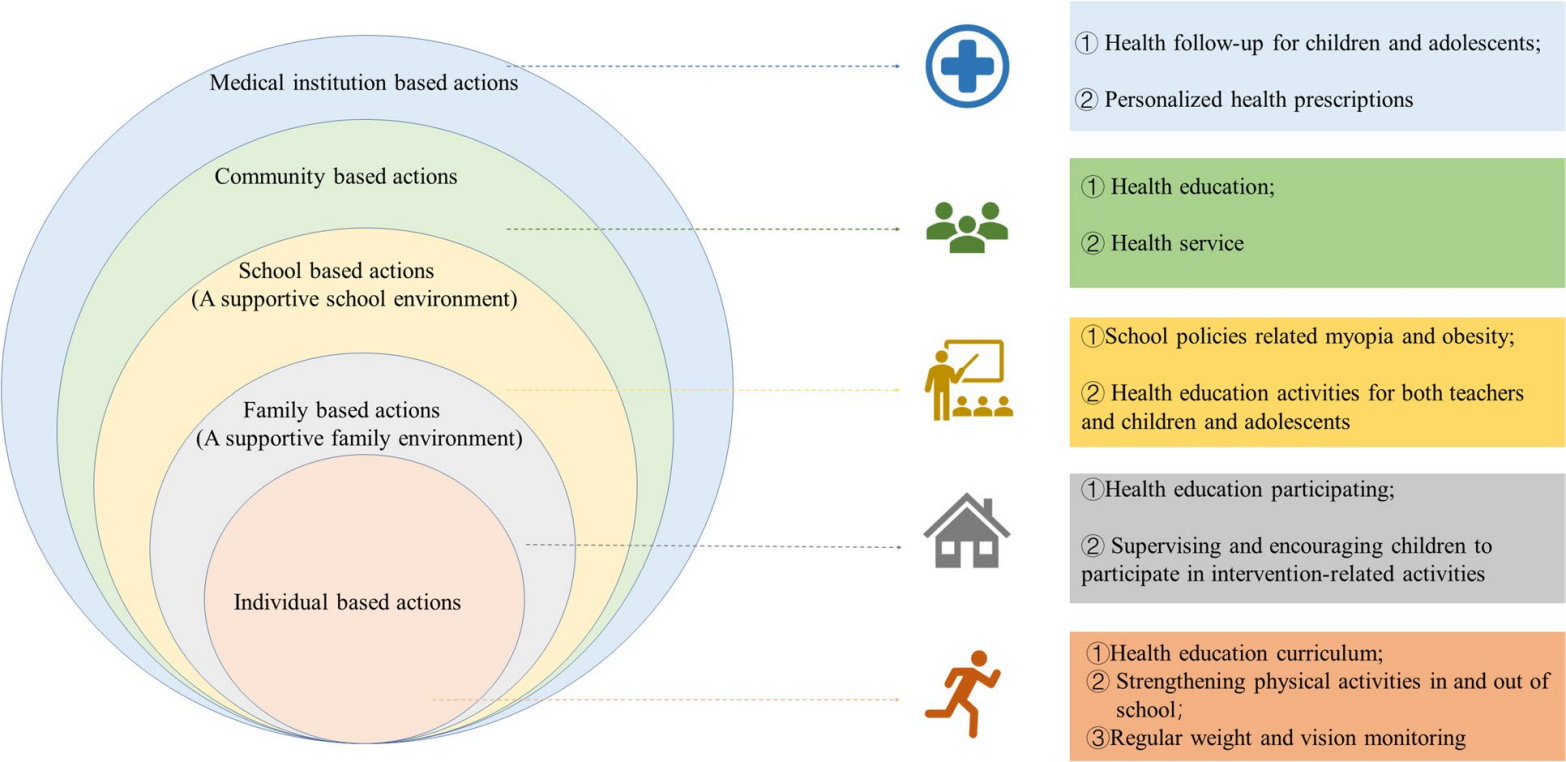
A social ecological model for the interventions designed across various personal and environmental factors influencing child and adolescent obesity and myopia

**Fig. 3:**
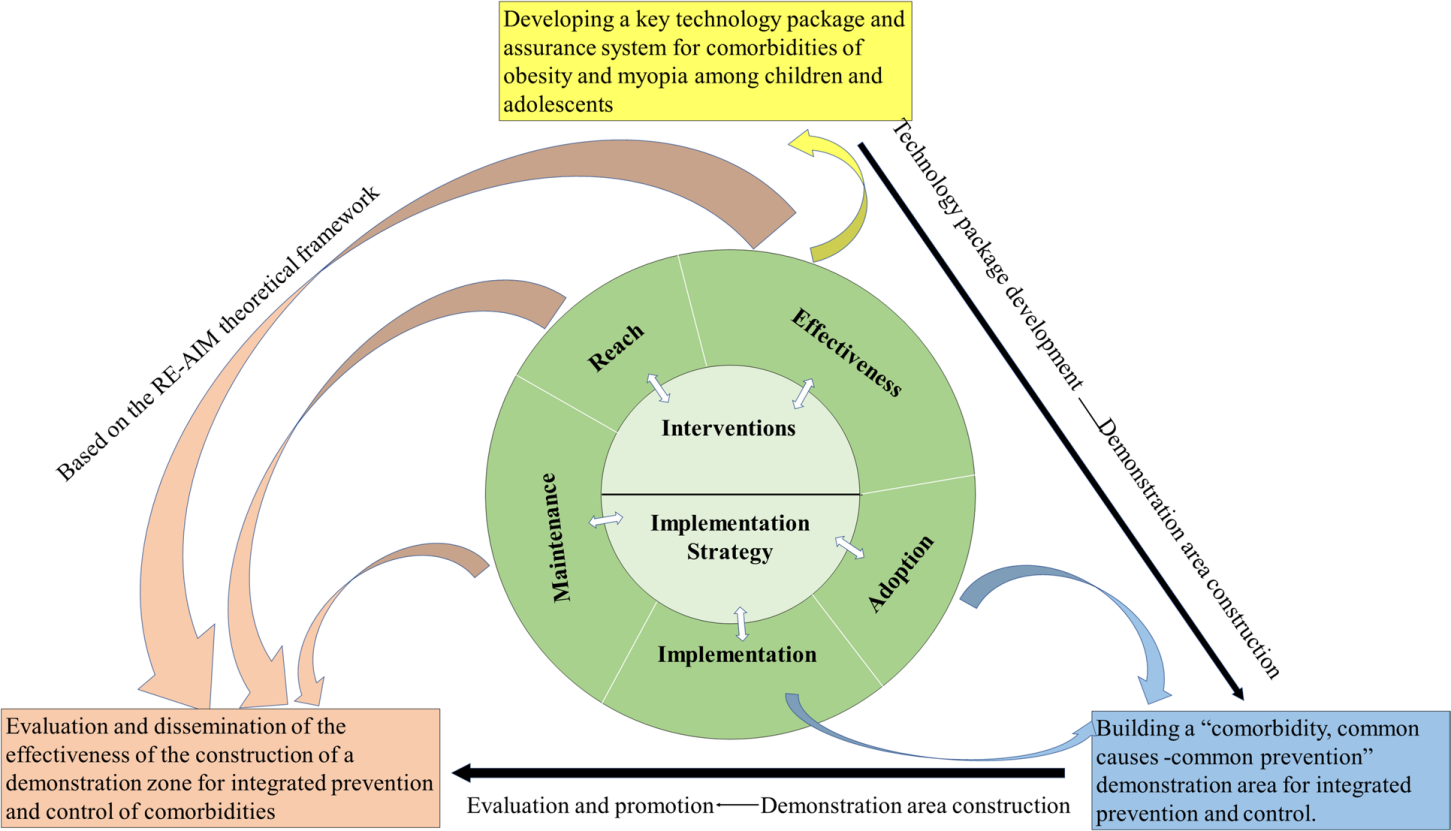
The study design under the RE-AIM framework

This is a hybrid type I effectiveness‑implementation trial with two stages (Fig. 1). The first stage is to develop a comprehensive SEM-based intervention strategy for myopia and obesity prevention in children and adolescents through evidence-based, technology development and technology integration, and to assess its effectiveness (Fig. 2). The second phase is to evaluate the implementation with regard to acceptability, feasibility, adoption, usage, and maintenance (Fig. 3). This study protocol adhered to the SPIRIT guidelines for clinical trial protocols [50] (Supplementary Material).

### Effectiveness evaluation

#### Study design

**Fig. 4:**
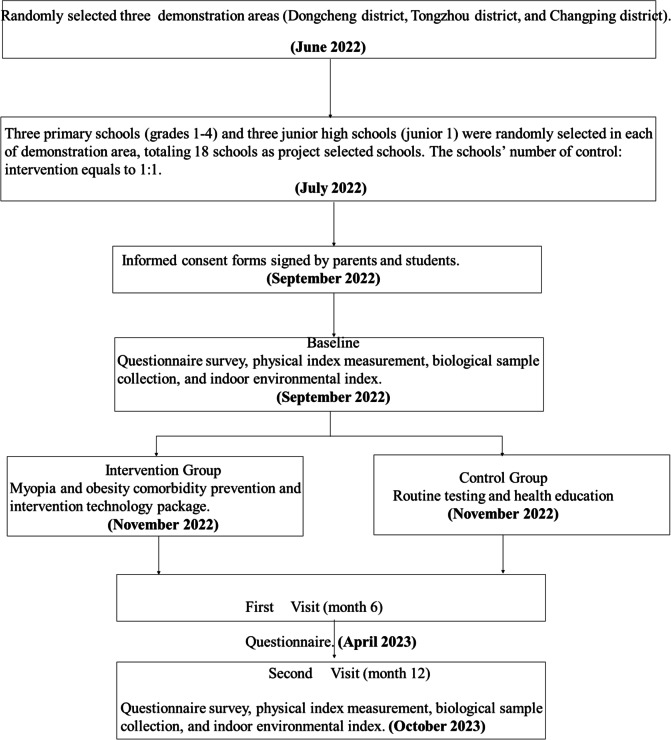
The flow chart of participants in the trial

A two-arm cluster randomized trial with a parallel-group of a 1:1 allocation will be conducted in three districts (Dongcheng District, Tongzhou District, and Changping District) according to their geographical location and socioeconomic conditions in Beijing and then selected schools in the districts for a 12- month intervention. In each of the three districts, 3 primary schools (grades 1–4), 3 junior high schools (junior 1), and a total of 18 schools will be selected as intervention schools, while other 18 schools will be regarded as control schools. t. Children and adolescents grades 1–4 and 7 with aged 6–14 years will be recruited for advoiding missing follow-ups due to further education into other schools (Fig. 4). All intervention schools in the three districts will use a standardized and uniform research protocol.

Given the evidence-based research and qualitative survey, we will discuss the development of an intervention model for myopia and obesity co-morbidity, optimize the relevant techniques, integrate multi-dimensional indicators through experts and school fronteirs meetings, as well as pilot study for children and adolescents and their families, and then establish an accurate assessment system for the health risks of myopia and obesity in children. A social-ecological intervention model with the participation of “individual, family, school, community, and clinic” centered on individual children and adolescents will be established.

### Recruitment

#### Recruitment of the schools

For the schools participating in the program, the size of a class was varying from 30 to 50 children and adolescents per class. If the number of children and adolescents in one class is less than 40, we will recruit two classes from the corresponding grade, and if the number of children and adolescents is greater than 40 of one class, we will recruit one class. School inclusion criteria are: 1) School principals should agree and cooperate with the project team to implement the intervention plan, and the school has health related human resources itself, such as school doctors, health teachers, and physical education teachers; 2) The total number of children and adolescents in each grade should be larger than 50. School exclusion criteria are: 1) Boarding schools and specialty schools for children and adolescents with talents or minority ethnic groups will be excluded; 2) Schools will also not be included if they have a definite plan for relocation or cancellation in the next 1 year.

Four steps will be applied for the recruitment of the schools. First, the education authority of each district will be contacted through a project workshop to seek their input on the study proposal. Second, the education authorities of each district will provide basic information on the participating schools (school type, number of children and adolescents, and teachers). Third, project team will hold school seminars to understand the specific situation of the school, conduct field visits to the school, and finally determine the selected school for the project. Fourth, the final list of eligible schools and classes will be determined by the principal investigator, and schools will be invited to participate in the study by the project partners.

#### Recruitment procedure of the study population

Within one week before the start of the recruitment process for study participants, the project team will distribute informed consent forms to all children and adolescents in the selected class through their classroom teachers, and the forms will be taken to home for their parents to complete (Fig. 4). Only children and adolescents who meet the inclusion criteria and provide signed individual and parent informed consent will be invited to participate in the project. The inclusion criteria are: (1) Grades 1–4 or 7 with ages 6–14; (2) Complete physical examination data at school; (3) No transfer plan within one year. Children and adolescents will be excluded if parents report one of the following: (1) A history of heart disease, hypertension, diabetes, tuberculosis, asthma, hepatitis, or nephritis; (2) Obesity due to disease or drug side effects; (3) Abnormal physical development, such as dwarfism or gigantism; (4) Physical deformities, such as severe scoliosis, chicken breasts, lameness, marked O-leg or X-leg; (5) Unable to participate in school sports activities; (6) Weight loss due to vomiting or medication in the past three months; (7) Ophthalmic device diseases, such as keratitis, corneal ulcer, etc.; (8) History of eye surgery, such as laser surgery, etc.; (9) Visual problems such as color weakness and color blindness.

### Intervention

#### Intervention group

In the context of school-centered multifaceted interventions, we will establish a gradient prevention and control system based on the SEM, with the “22510SS” comprehensive intervention strategy as the core. The aim is to mitigate and manage myopia and obesity among children and adolescents through targeted interventions at the individual, family, school, community, and clinical levels. Table 1 provides an overview of the intervention process.

**Table 1:** Participant timeline according to SPIRIT

	**STUDY PERIOD**			
	**Enrollment**	**Allocation**	**Post-allocation**	**Close-out**
Time point	September–October 2022	October 2022- April 2023	June-July 2023	November–December 2023
**Enrollment**				
Eligibility screen	X			
Informed consent	X			
Allocation (cluster)		X		
**Interventions**				
Intervention		X		
Control		X		
**Assessments**				
Anthropometric measures	X			
Biospecimen measures	X		X	X
Environmental measurements	X		X	X
Questionnaire	X			

The “22510SS” package will primarily be implemented in 18 intervention schools and extended to families as well as nested community and clinics into the worknet by project team. To support this, individual early warning information and corresponding intervention plans will be disseminated via a self-developed WeChat applet. Data collection and intervention will be conducted through an “individual, family, school, community, and clinic” data-sharing module centered on children and adolescents. The project team will work with clinical doctors and school health providers to utilize the WeChat applet to facilitate quality control, regular supervision, follow-up, lifestyle adjustment, and implementation of intervention plans, with timely feedback to families and schools for continued implementation. Clinical doctors will help identify the children and adolescents with different level of risk on myopia or obesity. For high-risk group, including children with rapid progression of myopia, severe visual health, and moderate to severe obesity or other metabolic diseases, physicians in pediatrics, ophthalmology, and endocrinology will provide further diagnosis and prescribe healthy diets and exercise; For potential risk groups strengthen supervision and provide relevant health services; For low risk groups strengthen health education and enhance health concepts. Regular tracking and feedback intervention will be provided, with information shared with individuals, schools, and families through the self-develoed WeChat applet, and families, schools, communities, and clinical will jointly implement intervention prescriptions.

Intervention measures will be taken against children and adolescents’ myopia and obesity at four levels: individual (individual centered activities), environment (supportive family and school settings), and supervision (family, school, program team, and clinical) and feedback (timely feedback from the WeChat applet). Table 2 provides a detailed overview of the specific intervention concepts.

**Table 2:** The key messages of prevention and intervention for the comorbidity of myopia and obesity

Intervention Concept	Messages
2 (2 h daytime outdoor activities)	1. More than 2 h of daytime outdoor physical activity every day, with at least 1 h moderate to high-intensity daytime outdoor physical activity
2 (Limit screen time to no more than 2 h per day)	2. The screen usage time should not exceed 2 h, of which the recreational use of electronic products should not exceed 1 h per day, and each time should not exceed 15 min
5 (Consume at least 5 servings of fruits and vegetables daily)	3. Eat 5 servings of vegetables and fruits the size of an adult’s fist, about 300 to 500 g of cooked or chopped lettuce leaves, and about 200 to 400 g of fruit (to ensure the supply of fresh vegetables and fruits, it was recommended to choose vitamins rich in vegetables and fruits of A and C, such as spinach, leeks, pea shoots, alfalfa, green peppers, sweet potatoes, carrots, pumpkin, etc.)
1 (Attain 1 h of physical activity daily)	4. Eat 1 serving of lean meat every day, which was equivalent to the size and thickness of the palm of an adult, about 80 to 110 g. It was recommended to choose high-quality proteins rich in vitamin A and zinc, such as beef and fish
0 (Consume 0 sugar-sweetened beverages)	5. Avoid sugary drinks
S (Reasonable sleep duration)	6.1 Adequate sleep time: 10 h in primary school and 9 h in junior high school. 6.2 Good sleep habits: Go to bed late, get up early, get up early, go to bed early, don’t stay up late, and don’t do anything unrelated to sleeping in bed
S (Regular supervision)	7.1 Realize the functions of knowledge dissemination, goal setting, behavior monitoring, evaluation feedback, and encouragement and guidance through the application of the official account. 7.2 Build a visual and physical index collection and management system, dynamically monitor students' visual acuity and obesity-related indicators, conduct assessments sent, and achieve timely early warning. 7.3 Make a handbook for the prevention and control of myopia and obesity comorbidity, teach students and parents teachers, train little guards for comorbidity prevention, and enhance students’ sense of ownership

##### Individual focused activities

Will include health education activities for children and adolescents, enhanced their physical activity in and out of school, and regular supervision of vision, weight, and height. Seen Table 3 for details.

**Table 3:** Intervention actions under a socio ecological model

Content	Frequency and duration	concrete measure	Executives
1. ***Individual focused activities***			
Health education activities	Health education curriculum (40 min each) would be held every 2–3 weeks, 6 classes per semester, 12 classes in total	The health education curriculum will be to focuses on disseminate health knowledge and consolidating key messages through interaction, games, and summaries 1) Key messages will include the benefits of healthy weight and vision, measurement and assessment of weight and vision, and ways to achieve healthy weight and vision (22510S). Health education books would be distributed to children. Health education messages would be disseminating on campus or in classrooms in the form of posters and brochures. 2) After each health education campaign is disseminated, we will be arranged an after-school daily punch card activity (e.g. a three-day challenge to get off the electronic screen). 3) Feedback will be provided on the results of regular monitoring of children’s BMI and behavior, with encouragement given to children who do well	Medical institution and selected schools
Sports activities in schools	2 h or more of outdoor physical activity per day, including at least 1 h of moderate to vigorous physical activity	Physical education teachers instruct children in outdoor activities (including physical education, recess, and extra-curricular activities) at school, include at least one hour of moderate to vigorous physical activity per day. If the school meets this requirement, no additional outdoor activities will be added to the school schedule le, otherwise, additional outdoor activities ((e.g. PE, recess, or extra-curricular activities) will be added to the school schedule. The implementation of these physical activities will be monitored on an ongoing basis during the intervention period;	Project partner and school physical education teachers
2. ***Family activities***			
Health education activities for parents	Health education activities will be held twice a term (60 min each) for a total of 4 sessions	1) Promote parents’ health literacy and create a positive family health environment, and will play a supervisory and guidance role in the implementation of the intervention. 2) The role of outdoor activities in preventing myopia, overweight and obesity and promoting physical and mental health will be publicized. Popularizing outdoor activities is not to emphasize the content and method of “activities” but to emphasize the positive significance of “outdoor”, so as to maximize the resonance of schools, parents and students.3) Help parents to learn about mobile phone microphones. 4) Parents will be helped to learn the functions of the mobile phone WeChat public website. 5) Project staff will provide feedback to parents on their children’s vision and weight status and behavior and will meet and discuss with them face-to-face	Medical institution and selected schools
Sports activities outside of school	30 min/day on weekdays and 2 h/day on weekends	PE teachers instruct parents on how to lead their children in outdoor activities outside of school on weekends (including PE, recess, and extra-curricular activities), include 2 h of outdoor exercise on weekends, with at least one hour of moderate-to-vigorous physical activity. Incentive policies are also in place, such as an exercise punch card with prizes. Parents will be encouraged to walk to school or accompany their children on walks to school, and spend more time with their children on weekends and holidays to participate in outdoor activities, get out into nature and be close to the sun. The implementation of these out-of-school physical activities will be continuously monitored by project staff during the intervention period	Project partner and school physical education teachers
3. ***School activities***			
Creating a healthy environment in schools	Throughout the intervention period	1) School will be provided with guidance on interventions and recommended policies for schools to implement, such as not selling, eating, or buying unhealthy snacks or sugary drinks on school premises, ensuring that health education programs and school physical education classes are implemented properly, and posting posters on school bulletin boards to create a positive atmosphere. 2) The school will plan to y implements the policy requirements of “sunshine physical activity” for students and the mastery of 1–2 physical skills by each student, improves the school environment, increases recess time in the morning and afternoon, and encourages teachers and students to participate in outdoor activities together. Students in the lower grades can be led by their teachers to play group games, while students in the upper grades can be free to jump rope, play shuttlecock, run, play basketball, etc. in the playground	Project partner
Health education activities for school teachers	One health education activity (60 min each) will be held once a term for a total of 2 sessions. School teachers (headmasters, classroom teachers, school nurses, and physical education teachers) from each school are required to participate in this activity	1) To improve teachers’ health literacy, create a good school health climate and act as a supervisor and guide in the implementation of the intervention. 2) Teachers will be helped learn the functions of the mobile phone WeChat public website. 3) Project staff will provide feedback to teachers on children's weight status and behavior and have face-to-face meetings and discussions with teachers	Medical institution and selected schools
4. ***WeChat applet***			
Information dissemination	Timely updates	The mobile phone is WeChat. WeChat will provide health information to parents, class teachers, and project staff based on health education activities	Project staff
Behavioral monitoring	Once every one month	Parents and students will be asked to record their students’ eating and physical activity behaviors every week on WeChat and personalize the information related to these behaviors for feedback	Project staff and parents
Health Management	Once every one month	Parents, school teachers, and project staff will provide personalized feedback based on monthly monitoring of children’s vision, weight, and height, and changes compared to children’s previous records	Project staff, Parents, and school teachers
Assessment and feedback	Once every one month	Comprehensive and individualized assessments will be provided that incorporate changes in the child’s behavior and weight status	Project staff

##### Family activities

Will be toward to parents and include health education activities for parents, as well as supervision and encouraging children and adolescents to increase their levels of physical activity outside of school. Table 4 for details.

**Table 4:** Interventions for students

Intervention Concept	Interventions
2 (2 h daytime outdoor activities)	1. Go out of the classroom between classes and actively participate in large recess activities
	2. Put some indoor activities outdoors during the day, and actively participate in daytime outdoor sports interest groups
	3. Walk to school, set up a school group with classmates (“healthy school bus”), delineate the route for students to walk to and from school, and parents pick up and drop off children at specific times and specific stops
	4. Complete the homework assigned by the school for daytime outdoor activities, such as adhering to daytime outdoor physical activity for more than 2 h on rest days
2 (Limit screen time to no more than 2 h per day)	5. The single use of entertainment electronic products should not exceed 15 min, and the cumulative use of electronic products should not exceed 1 h per day. After 30–40 min of learning with electronic products, you should take a break and relax for 10 min. The younger you are, the shorter you should continue to use electronic products
	6. Avoid bad screen habits, do not use electronic products in dimly lit environments, and avoid using electronic products in a lying position and before going to bed
	7. When viewing the screen, the shoulders should be kept relaxed, the upper back should be extended, the upper arm should be at 90° to the forearm, the wrist should be relaxed, and breathing regularly
	8. When writing homework, keep your eyes about one foot away from the book (about 30 cm), your body about one punch (about 6 cm) away from the desk, and your fingers about one inch (about 3 cm) away from the tip of the pen
5 (Consume at least 5 servings of fruits and vegetables daily), 1 (Attain 1 h of physical activity daily) and 0 (Consume 0 sugar-sweetened beverages)	9. Take health education courses on dietary nutrition and develop a reasonable diet and healthy living habits
	10. Learn about food nutrition labels and choose food reasonably
	11. You should maintain balanced nutrition, not picky eaters, not partial eclipse, not overeat, and eat more fresh vegetables and fruits
S (Reasonable sleep duration)	12. Students should kept enough sleep every day. It was recommended that pupils should not be less than 10 h and junior middle school students should not be less than 9 h
	13. Avoid going to bed late and getting up late on an irregular schedule

##### School activities

Will included school policies related to the prevention of myopia and obesity and health education activities for teachers. Table 5 for details.

**Table 5:** Interventions for schools

Intervention Concept	Interventions
2 (2 h daytime outdoor activities)	1. Parents should create a family atmosphere of physical activity for children, and allow children to spend 1 h outside school every day
	2. Encourage students to walk or accompany children to school on foot
	3. Parents regularly monitor children’s growth and development, measure height, weight, and waist circumference at least every three months, and keep records
	4. Spend more time on weekends and holidays to participate in daily outdoor activities with children, walk into nature, and get close to the sun
2 (Limit screen time to no more than 2 h per day)	5. Control the time that children use electronic products. The daily entertainment video time should not exceed 20 min each time, and the total time should not exceed 1 h. Younger children and teens should have less screen time for entertainment
	6. Parents should regularly monitor their children’s vision. If they find that their children squint, rub their eyes frequently, or look at the text on the blackboard or objects in the distance that is unclear in class, they should consider the possibility of myopia and take their children to the hospital in time
	7. Instruct children to develop good eye habits, improve children's awareness of eye care and eye protection, instruct children to develop good eye habits, and avoid using eyes at close range for a long time
	8. Guide children to develop the habit of resting between their eyes for a long time, especially resting their eyes through exercise
5 (Consume at least 5 servings of fruits and vegetables daily), 1 (Attain 1 h of physical activity daily), and 0 (Consume 0 sugar-sweetened beverages)	9. Buy food as needed, choose fresh and hygienic food, and store it reasonably
	10. Learn about food nutrition labels, pay attention to food labels when purchasing food, and choose healthy food for students
	11. Parents provide students with small portions and a variety of food, encourage them to eat more vegetables, fruits, and high-quality protein at home, eat fewer sweets, and not drink sugar-sweetened beverages. Ensure regular meal times, meal locations, and appropriate eating or serving utensils. Supervise to help students eat slowly, not picky eaters, not partial eclipse
S (Reasonable sleep duration)	12. Implement appropriate restrictions on students’ use of electronic products before bedtime
	13. Lead by example, consciously maintain a good routine, let students recognize the importance of sleep, and develop a good habit of independently maintaining a routine

##### WeChat applet

Will be provided to project team, clinic doctors, school teachers, and parents to follow. The applet will develope corresponding application modules based on behavior change technology. Assistance will be provided in information dissemination, behavioral supervision, weight management, assessment, and feedback. Table 6 for details.

**Table 6:** Interventions for parents

Intervention Concept	Interventions
2 (2 h daytime outdoor activities)	1. Under the guidance of the teacher, let each student master 1–2 physical skills
	2. Improve the school environment, increase the large class time in the morning and afternoon (one in the morning and one in the afternoon, 30 min each time), and encourage all teachers and students to participate in daytime outdoor activities together
	3. Encourage students to go out of the classroom between classes. The lower-grade students can be led by teachers to play some group games, etc.;
	4. Guarantee the school physical education class and the big break time: the school should try to avoid the phenomenon of physical education class being occupied or suspended, and if the weather is bad, it should organize students to exercise in indoor venues
2 (Limit screen time to no more than 2 h per day)	5. Control the teaching time of using electronic products in schools, in principle, it does not exceed 30% of the total teaching time
	6. Teach children the knowledge and skills of weight control and myopia prevention through classroom lectures, visits and demonstrations, face-to-face tutoring, and group activities
5 (Consume at least 5 servings of fruits and vegetables daily), 1 (Attain 1 h of physical activity daily), and 0 (Consume 0 sugar-sweetened beverages)	7. School canteens or catering companies provide students with food that is nutritionally balanced, reasonably cooked, and in line with local dietary characteristics, provide nutrition labels for catering food, and feedback on the appropriate recipes to parents
	8. In the whole school, the school canteen, commissary, vending machine, and other places eliminate the supply of unhealthy food such as sugar-sweetened beverages, potato chips, and sweets; unhealthy foods such as sugar-sweetened beverages, potato chips, and sweets are not allowed in the school. Educate students not to buy unhealthy foods such as sugary drinks, potato chips, and sweets at school gates
	9. Regularly post health education posters and improve school food environment signs on the notice board on campus and in the classrooms of participating classes
S (Reasonable sleep duration)	10. Establish reasonable school hours for students
	11. Reduce the burden of homework after school, and do not let too many academic tasks crowd out students' sleep time

#### Control group

The children and adolescents in the control schools will have only regular monitoring and class meetings or health education classeswithout clinic doctors’ paricipating.

### Quality control of the intervention

Two manuals have been developed to facilitate the implementation and management of this complicated intervention. These manuals include the “Operation Manual for Intervention Project Team members” and the “Operation Manual for Intervention School Team Members”. The manuals provide the specific technologies, frequency, and duration of the intervention, as well as a checklist to ensure that the intervention is implemented as required. The manuals also outline the training and responsibilities of each delivery person involved in the project, such as project members, school principals, school doctors, health teachers, physical education teachers, as well as children and adolescents. Additionally, the manuals provide detailed instructions regarding the responsibilities and division of labor of project team and school team members, for the implementation of intervention measures. They also describe the workflow for each intervention content, outlining who, when, how, and to what extent the specific intervention content should be delivered. All project team and school team members are expected to adhere to the operational manuals in the delivery of intervention. The intervention implementation process will be monitored regularly, and feedback will be provided by project team in a timely manner.

### Data collection

#### Qualitative data collection

##### Expert interview

Has been conducted by individual interview or focus group for intervention package development and format “22510SS”. The experts and scholars are senior and experinced in the field of child and adolescent health. The interviews has been organized by trained investigators.

##### Delphi study

Has been conducted with 20 experts from various fields, including ophthalmology, endocrinology, pediatrics, school health, school sports, health education, and stakeholders with extensive experience in the prevention and control of children’s myopia or obesity. The study has carried out three rounds of expert consultation to determine intervention package and make the priority order of intervention package via email.

#### Quantitative data collection

In March 2023, we will conduct baseline data collection and assessments for both the intervention and control groups. Throughout the 12-month intervention period, we will conduct periodic follow-up assessments as outlined in Table 7. Anthropometric data, biospecimen data, and environmental measurements will be collected at baseline and at follow-up at the end of the intervention. All measurements will be taken uniformly trained and qualified staff and collected by investigators using the same equipment and/or forms by standard methods and procedures. Investigators who measure children and adolescents’ visual acuity, refraction, height, and weight will be blind to the information of intervention or control group. Table 8 shows a trial participant timeline.

**Table 7:** Interventions for schools

Intervention Concept	Interventions
S (Regular supervision)	1. The project team regularly disseminates the relevant knowledge of the project’s health education activities to students, parents, and teachers through the mobile WeChat official account
	2. Parents and students fill in a health behavior questionnaire on a regular basis through the WeChat official account of the mobile phone. The project team and the school can learn about the health behavior of students through the official account
	3. The project team will automatically generate comprehensive evaluation results based on the student's physical examination information and WeChat official account
	4. The project team uses the mobile phone WeChat official account to dynamically monitor the vision, height, and weight of all students participating in the project. The mobile phone WeChat official account automatically provides encouragement or guidance to students, parents, teachers, and the project team
	5. Parents upload the student's physical examination or weight monitoring information every three months to the mobile WeChat public account. The mobile WeChat public account will provide health prescriptions issued by experts from Beijing Children’s Hospital Affiliated with Capital Medical University, and provide information on intervention and treatment. Timely feedback is provided to individual families and schools, and intervention prescriptions are jointly implemented by home schools and the community

**Table 8:** Contents of the baseline survey and secondary follow-up survey

Measurement indicators	Baseline survey	3 month follow up	12 month follow up	Method
***Anthropometric measures***				
Height	✓	✓	✓	Measured to the nearest 0.1 cm at least twice
Weight	✓	✓	✓	Measured to the nearest 0.1 kg at least twice
Waist and hip circumference	✓	✓	✓	Measured to the nearest 0.1 cm at least twice
Blood pressure	✓	✓	✓	Measured to the nearest 1 mm Hg. The average of the last two of three measurements
Visual acuity	✓	✓	✓	The distance vision test must be performed in both eyes separately, the right eye first and then the left eye
Diopter	✓	✓	✓	The test should be performed using a desktop automated computerized optometrist and should be averaged 3 times per eye
Body composition	✓	✓	✓	The body composition analyzer adopts a 4-point contact electrode for measurement
***Biological samples***				
Blood	✓	-	✓	Collect 5 ml of venous blood
Saliva	✓	-	✓	Collection of 10 ml of mid-stream urine
Urine	✓	-	✓	Collect 5 ml of saliva
***Measurement of indoor environmental indicators***				
Household air pollution exposure	✓	-	✓	Continuous measurement with illuminometer for 7 days
Night light exposure	✓	-	✓	7 consecutive days of measurement with sensors
***Questionnaire***	✓	-	✓	
Student Questionnaire	✓	✓	✓	Issues related to children and adolescents
Parental Questionnaire	✓	✓	✓	Issues related to parents and children
School Questionnaire	✓	✓	✓	School related issues

Anthropometric measures includes visual acuity, refraction,height, weight, waist circumference, hip circumference, blood pressure, blood routine, and body composition, and 5% of the samples were randomly selected for morphological index analysis measurement.

Biospecimen measures consisits of blood, urine of saliva. All biological samples were collected after fasting for 12 h, and biochemical analysis of all biological samples was performed by a biomedical analysis company accredited by Peking University.

Environmental measurements household air pollution exposure and nighttime light exposure.

Questionnaire will be utilized to assess the behaviors of children and adolescents, as well as school policies for the prevention and management of myopia and obesity, along with other possible moderators/mediators of intervention. The questionnaire has been developed based on previous research and pilot studies, and is deemed feasible for this study, as well as acceptable to both children and adolescents and their parents. For children in grades 1–4, parental assistance will be provided to complete the questionnaire, whereas students in grade 7 will receive guidance from their class teacher when filling out the questionnaire.The parent questionnaire will focus on parental information and health behaviors, while the school questionnaire will cover basic information, myopia and obesity intervention work, personnel management, health education, sports activities, nutrition, and classroom environmental sanitation supervision. The trained project members will provide detailed explanations of the questionnaires, and assistance and guidance will be given as needed to complete them effectively. The project team staff will review 3% of the questionnaires within one week.

### Outcomes

#### Primary outcome

The primary outcome of this study is to assess the differences in changes in visual acuity and body mass index between children and adolescents 12-month after baseline as evaluated by medical examination. Between-group differences in changes in outdoor activities screen time, fruit and vegetable intake, high-quality protein intake, sugar beverage consumption, sleep duration, and supervision will be assessed using questionnaires.

(‘2’). Engage in at least 2 h of outdoor physical activity during the day, with at least 1 h of moderate to high intensity exercise.

(‘2’). Limit screen time to no more than 2 h per day.

(‘5’). Consume 5 servings of fruits and vegetables, each equivalent to an adult’s fist, daily.

(‘1’). Consume 1 serving of lean meat, equivalent to an adult’s hand, daily.

(‘0’). Avoid sugary drinks.

(‘S’). Ensure adequate sleep, with elementary school children needing 10 h and junior high school students needing 9 h of sleep each night.

(‘S’). Parents and students should provide timely feedback and cooperate with efforts to prevent and control myopia and obesity.

#### Secondary outcomes

Secondary outcomes will be evaluated using the RE-AIM framework to comprehensively assess the acceptability, feasibility, adoption, usage, and maintenance of the intervention study.

(‘Reach’). We will assess the reach of the program by measuring the number of individuals who expressed willingness to participate,

(‘Effectiveness’). The effectiveness of the intervention, including why and how it worked, or did not work.(‘Adoption’). We will examin the adoption of the program by students, parents, and schools, as well as the likelihood and reasons for willingness to accept the intervention in the future.

(‘Implementation’). Whether the elements of the intervention were actually accepted by the study participants and the school as an indication of whether the achievement of the desired outcomes of the myopia and obesity intervention was due to the content of the intervention itself or the way in which the intervention was implemented.

(‘Maintenance’). We will evaluate the maintenance of the intervention by analyzing how well the behavior was maintained after the intervention ended and whether it became a daily practice and policy in the school.

### Sample size

In our study, we aimed to investigate the co-morbidity of myopia and obesity among Beijing children and adolescents. Based on previous studies, the co-morbidity rate was expected to be 17.3% in the intervention group and 13.5% in the control group, setting a two-sided α = 0.05 and a 90% certainty. Using the PASS15 software, the sample size N1 = 1891 cases for the intervention group and N2 = 1891 cases for the control group were obtained according to the formula $( {\text{n}}=\frac{2\overline{p }\overline{q }{\left({\text{Z}}_{\alpha }+{\text{Z}}_{\beta }\right)}^{2}}{{\left({\text{p}}1-{\text{p}}2\right)}^{2}} )$ to the randomized controlled trial, taking into account the loss and refusal of 20% of the cases, at least 2250 cases each for the intervention and control groups were finally required, and at least 4500 cases were included in total.

### Statistical analyses

For the qualitative data, we will use Excel 2016 for data input and SPSS 25.0 for statistical analysis. Statistics will include score mean, standard deviation, coefficient of variation, etc. The key basis will be whether it will be used as a common intervention concept for the prevention of comorbidities. Experts will score items with a mean greater than 3.5 and a coefficient of variation less than 0.25. Items will be selected, and the item descriptions will be modified regarding expert suggestions.

For quatitative data, we will double entere by using Epidata 3.1 analyze using SPSS 25.0. The primary outcome will be group differences in changes in visual acuity and body mass index in children and adolescents at 12-month after the baseline. We will use a mixed-effects model to compare the group difference, taking school into account due to its cluster effect. Then we will conduct subgroup analysis to identify the interaction effect (stratification factor × group) and analyze the differences in intervention effects between strata. Furtherly, we will use difference-in-difference analysis to analyze differences between the intervention and control groups before and after the implementation of the intervention. The secondary outcomes will be analysed in the similar steps. All statistical tests will be two-sided at the 5% level of significance.

### Implementation evaluation

#### Study design

Given that evidence-based interventions and practices are poorly implemented, and might take up to many years to adopt and integrate the interventions and practices into routine work by practitioners and policy-makers [51, 52], to close the know-do gap and accelerate the implementation, We will adopt the RE-AIM that evaluates implementation strategies through implementation outcomes, including the five dimensions of Reach, Effectiveness, Adoption, Implementation, and Maintenance into this trial. Therefore, we identify this study as a type 1 hybrid design implementation study to determine the effectiveness and explore the context of routine implementation [53].

#### Process evaluation

Based on the steps and principles described in the conceptual framework proposed by Saunders etc. [54], we will identifyand evaluate the following process evaluation elements: 1) Fidelity (quality): the extent to which the intervention will be implemented as initially planned. 2) Dose delivered (completeness): the frequency and intensity with which the program will be implemented. 3) Dose received (exposure): the extent to which children and adolescents, parents, and teachers were exposed to the intervention. 4) Dose received (satisfaction): student, parent, and teacher satisfaction with interventions and materials. 5) Reach (the proportion and characteristics of children and adolescents, parents, and teachers who completed or dropped out of the intervention) and context (the family environment and school policies related to myopia and obesity prevention and management).

The implementation process evaluation will mainly include: 1) Regular statistics on the number of hits and feedback on the intervention sessions on the WeChat applet. 2) User logs (e.g., frequency and duration), which will be collected by the WeChat app. 3) Regular qualitative interviews with intervention school family committee mothers and classroom teachers. 4) School policies related to myopia and obesity prevention and management, will be collected through a school questionnaire.

### Statistical analyses

To analyze the five dimensions of myopia and obesity intervention for children and adolescents in Beijing under the framework of RE-AIM. Reach will be calculated based on the proportion of eligible participants in the intervention among children and adolescents, families, schools, communities, and healthcare facilities. Effectiveness will refer to the effect at the individual level of children and adolescents after the intervention will be implemented, mainly including biochemical indicators, physical indicators, indicators related to health knowledge and behavior, and myopia and obesity reporting outcomes. As well as related indicators for households, schools, communities and healthcare facilities. Adoption will be calculated based on the proportion of families, schools, communities, and medical institutions that implement intervention measures. Implementation refers to the implementation of the project by schools and doctors, the cost of project implementation, and the adjustment and improvement in project implementation. Maintenance will refer to the continued implementation of the intervention by schools and families, and will continue participation of children and adolescents after the intervention is over.

## Discussion

This study aimed to develop a comprehensive intervention strategy “22510SS”, to combat the prevalence of myopia and obesity among children and adolescents, guided by a SEM and evidence-based interventions. The strategy involved interventions in individual, family, school, community, and clinic, and its effectiveness and implementation will be evaluated within the RE-AIM framework. If the study works, it will be embeded into the regional monitoring system in Beijing, to promote the development and evaluation of comorbities prevention services for children and adolescents, and promote the establishment of a healthy and friendly environment system for children and adolescents.

Although there have been numerous individual intervention studies on myopia and obesity in children and adolescents, there is currently no comprehensive intervention plan addressing both conditions. Our project team has optimized and integrated key technologies developed in the early stages to prevent myopia and obesity in children and adolescents, and apply these technologies in the Dongcheng, Tongzhou, and Changping districts of Beijing through pre-testing. This enables us to develop an evidence-based intervention of “22510SS” to use in this trial.

Despite the factors influencing myopia and obesity in children and adolescents are complex, they are primarily distributed across individual, family, school, community levels, and clinics [55, 56]. Previous intervention studies have mostly focused on school or family-based approaches, while ignoring the impact of community and medical institution interventions on children and adolescents with myopia and obesity [57, 58]. The development and assessment of interventions targeting myopia and obesity have been carried out through expert workshops and expert letters, followed by pre-surveys involving 300 participants across 18 intervention schools. The interventions were designed to bring about change at multiple levels, including children and adolescents, families, schools, communities, and healthcare organizations.

Several systematic reviews [59] have highlighted the importance of intensity and measurement of daytime physical activity and screen time. As such, our interventions require children and adolescents to engage in more than 2 h of daytime outdoor physical activity and limit their screen time to no more than 2 h per day. We encourage them to participate in recess and develop an interest in sports, while also cultivating good screen usage habits. To dynamically monitor and improve the effectiveness of interventions, children and adolescents will upload data on indicators such as height, weight, and vision to a WeChat applet every one months. This will allow us to track progress and make adjustments as necessary. To reinforce key recommendations for eye use, diet, and exercise behavior, we emphasized an easy-to-remember slogan: “22510SS”. Simultaneous we have also taken into account the application and implementation of interventions for high-risk and diseased populations. Our population is classified into three groups: the general population, high-risk population, and diseased population. Health prescriptions are issued by professional medical institutions for high-risk and sick groups, including children and adolescents with rapid progression of myopia, severe visual health status, moderate to severe obesity, or other metabolic diseases. These health prescriptions will be jointly administered by individuals, families, schools, communities, and clinics.

Previous intervention studies have demonstrated satisfactory short-term intervention effects, but maintaining long-term and repeatable effects has been a challenge due to the termination of intervention projects. To address this, our study established an evaluation model based on the RE-AIM framework, which evaluates the feasibility, sustainability, fairness, potential impact, acceptability, health economics, and health economics of the intervention model in the demonstration area [60]. This continuous evaluation promotes the sustainability of intervention effectiveness and enables policies and interventions to be constantly improved and developed. Moreover, the experience of the demonstration area has been extended to all primary and secondary schools in Beijing through the “National Monitoring of Common Diseases and Health Influential Factors of Children and adolescents” system, increasing the influence of the intervention measures and the durability of their effects. Our approach can provide a valuable framework for promoting the long-term effectiveness of existing interventions and improving the health outcomes of school-aged children.

This study has several notable strengths. First, it represents the first comprehensive intervention in China aimed at reducing the risk of two common diseases in children and adolescents: myopia and obesity. The intervention strategy, known as “22510SS”, draws on the social-ecological model (SEM) and considers the roles of individuals, families, schools, communities, and healthcare providers for optimal efficacy. Second, the study will use a RE-AIM-based framework to evaluate the intervention strategy and implementation outcomes.While this study is expected to make significant strides in reducing the risk of myopia and obesity in children and adolescents, there are potential limitations to consider. For example, the study will only recruit volunteers from three districts in Beijing, which may not be representative of all Beijing children and adolescents, let alone the entire population of China. Furthermore, as a hybrid design implementation study, some of the results will be assessed primarily through qualitative interviews. Therefore, the feasibility, acceptability, and sustainability of the intervention in practice may not be fully represented. As a result, the generalizability of the study's findings may be limited.

Based on the discussion above, it could be concluded that this topic is expected to have a significant impact on promoting the prevention and control of myopia and obesity in children and adolescents in China. Our research fills a gap in this area and if comprehensive results are obtained, the intervention strategy developed through this study could be a reasonable approach for addressing the comorbidities of myopia and obesity in Chinese youth. This research has the potential to make a meaningful contribution to the field of intervention research in China.

## Supplementary Information

**Additional file 1.** SPIRIT 2013 Checklist: Recommended items to address in a clinical trial protocol and related documents*.

## Data Availability

The datasets generated and/or analysed during the current study are not publicly available due confidentiality provisions in the consent process, but are available from the corresponding author on reasonable request.

## Abbreviations

BMI: Body mass index
SEM: Social ecological model
